# Comparison of Milk Fat Globule Membrane (MFGM) Proteins of Chianina and Holstein Cattle Breed Milk Samples Through Proteomics Methods

**DOI:** 10.3390/nu1020302

**Published:** 2009-12-22

**Authors:** Leonardo Murgiano, Anna Maria Timperio, Lello Zolla, Silvia Bongiorni, Alessio Valentini, Lorraine Pariset

**Affiliations:** 1 Dipartimento Produzioni Animali, Università degli Studi della Tuscia, Viterbo, 01100, Italy; Email: bongiorni@unitus.it (S.B.); alessio@unitus.it (A.V.); pariset@unitus.it (L.P.); 2 Dipartimento Scienze Ambientali, Università degli Studi della Tuscia, Viterbo, 01100, Italy; Email: timperio@unitus.it (A.M.T.); zolla@unitus.it (L.Z.)

**Keywords:** milk proteomics, MFGM, cattle, lactation, MS/MS

## Abstract

Identification of proteins involved in milk production is important to understand the biology of lactation. Many studies have advanced the understanding of mammary function and milk secretion, but the critical molecular mechanisms implicated in milk fat secretion is still incomplete. Milk Fat Globules are secreted from the apical surface of the mammary cells, surrounded by a thin membrane bilayer, the Milk Fat Globule Membrane (MFGM), formed by proteins which have been suggested to be cholesterolemia-lowering factors, inhibitors of cancer cell growth, vitamin binders, bactericidal, suppressors of multiple sclerosis. Using a proteomic approach, we compared MFGM from milk samples of individuals belonging to two different cattle breeds, Chianina and Holstein, representative of selection for milk and meat traits, respectively. We were able to isolate some of the major MFGM proteins in the examined samples and to identify differences between the protein fractions of the two breeds. We detected differences in the amount of proteins linked to mammary gland development and lipid droplets formation, as well as host defence mechanisms. We have shown that proteomics is a suitable, unbiased method for the study of milk fractions proteins and a powerful tool in nutritional genomics.

## 1. Introduction

Bovine milk has a high significance in human nutrition and economy, yet the characterization of its minor protein fraction repertoire is not complete [1]. Besides the major milk proteins (*i.e.*, caseins, lactalbumin and β-lactoglobulin), bovine milk contains low levels of serum-derived proteins such as albumin [2,3], enzymes like plasmin, complement proteins [4] and immunoglobulins [5], growth factors such as the IGF (insulin-like growth factors) family [6] and lactoferrin, an iron binding protein that has been shown to have antimicrobial properties [7]. Other minor milk proteins are known to be present at elevated levels during colostrum secretion and during drying off [8,9]. It has been shown that milk protein composition changes throughout the phases of lactation [10]. Proteins associated with inflammatory reaction have also been detected in milk during infections [11]. A whole additional range of biologically active proteins and peptides have been identified in milk, some of which show antimicrobial properties [12].

Proteomics is a powerful tool to identify relevant proteins in milk. Identification of proteins associated with the various aspects of milk production can provide a baseline for new research relative to the biology of lactation. Most of the proteomic studies conducted so far on mammary epithelial cells, organelles, membranes, and on the secretion processes, are focused on breast cancer, rodent lactation, or both [13,14,15,16,17,18]. These studies advanced the understanding of mammary function and milk secretion. Recently, papers on bovine mammary proteomics have been published, conducting a survey of proteins expressed in milk fat globule membranes, and showing development changes during lactation phases [19,20]. However, our understanding of the critical molecular mechanisms implicated in milk fat secretion is incomplete [22,23]. 

Milk-fat globules originate near the basal region of the secretory cells as small droplets of fat. They migrate through the cytoplasm, gradually increasing in size, as the synthesis of triacylglycerol proceeds. These Milk Fat Globules (MFG) are secreted from the apical surface of the cell, surrounded by a membrane thin bilayer, the Milk Fat Globule Membrane (MFGM) [13,24]. MFGM are formed by a unique and quantitatively small subcategory of milk proteins (approximately 2–4% of total protein in human milk), the content of which is still largely unknown [13,14]. Given the nutraceutical and biological importance of these proteins, studies on MFGM have recently been increasing [24,25,26]. The MFGM is a rich source of membrane proteins, and applied proteomic analysis of these membrane proteins, has highlighted some of the possible signaling and secretory pathways used by the mammary gland [19].

MFGM glycoproteins seem to contribute to the prevention of pathogenic organisms infections, being able to act as specific bacterial and viral ligands in the stomach of newborns, to prevent the attack of the intestinal mucosa [26]. The diversity of the glycans found in MFGM is thought to enable the glycoproteins to perform this function in the acidic environment of the stomach [14,23]. It has been noted that some forms of gastric diseases such as peptic ulcer, chronic type B gastritis and gastric cancer can be attributed to the colonization of gastric mucosa by *Helicobacter pylori* [28]. Non-defatted and defatted MFGM preparations, given orally, caused equal healing effect on *H. pylori* infection of gastric mucosa in BALB/cA mice, leading to the conclusion that the major role in inhibition of *H. pylori* infection is played by the protein fraction of milk fat globule membranes [29,30]. The rich nutrient content of milk and the body temperature inside the mammary gland provide optimal growing conditions for microbes [1]. The gland must develop a robust host defence system to counteract this condition. One aspect of such a defence system is the secretion of antimicrobial peptides and proteins into milk [30], which assumes an antimicrobial and immunomodulatory active function in the digestive tract of the newborn [31,32]. In the mammary gland there is a variety of glycoransferases synthesizing the oligosaccharide moieties present on the milk glycoproteins, homologues to the cell surface pathogen receptors in the stomach and intestine, which are supposed to be able to inhibit infection by competitively binding the pathogens [33]. MFGMs are rich in secretory immunoglobulin A (s-IgA) and several non-antibody proteins [34], particularly, it has been pointed out that MFGM-associated glycoproteins show antibacterial properties [35,36]. Oligosaccharide chains can enhance binding of s-IgA or MFGM glycoproteins to pathogens [35,36,37,38] preventing their growth/attachment on the mucosal cell membranes, where they might cause infections or deposit toxins [39,40].

Several MFGM proteins have been suggested as cholesterolemia-lowering factors, as well as vitamin binders, bactericidal, inhibitors of cancer cell growth, and suppressors of multiple sclerosis [41,42]. One of the most abundant and relevant MFGM protein is butyrophilin, a protein having effects on modulation of the encephalitogenic T-cell response to myelin oligodendrocyte glycoprotein (MOG), related to human multiple sclerosis, recently reported in experimental autoimmune encephalomyelitis (EAE) [41]. 

It has been shown that a bovine MFGM component, likely of proteic origin, could inhibit *in vitro* purified *E. coli* β-glucuronidase, the enzyme involved in the intestinal degradation of glucuronides. The enzyme glucuronyl transferase has an important role in detoxification of many metabolites of endogenous and exogenous origin. Glucuronyl transferase neutralizes the toxic compounds in liver cells through the formation of glucuronides, subsequently excreted. Some bacteria in the gut express the enzyme β-glucuronidase, which is able to degrade the glucuronides. This leads to the release of toxic agents, some of which might be carcinogenic. Therefore, the consumption of MFGM could prevent colon cancer due to the presence of the inhibitor of β-glucuronidase [43].

The consumption of the MFGMs alone as a nutraceutical, as a dairy food, or the consumption of food products enforced by MFGMs, may have health benefits due to the presence of phospholipids, constituting almost 30% of the total MFGM lipids [44]. The three main MFGM phospholipids are sphingomyelin, which can exert anticarcinogenic activity [45], phosphatidyl choline, and phosphatidyl ethanolamine [46,47]. Phospholipids, including milk-derived ones, are able to affect numerous cell functions including absorption processes, molecular transport systems, development and growth, memory, stress responses, myelination in the central nervous system and development of Alzheimer’s disease [48,49,50,51]. It has been suggested that MFG (milk fat globules) can serve as a putative delivery system for fat-soluble vitamins, organic phosphates, drugs and anticarcinogenic microelements such as Selenium [10].

MFGMs have an effect on lowering the serum cholesterol level. This has been confirmed by the observation of a direct inhibitory effect of bovine MFGMs on hypercholesterolemia in the rat [52]. However, the influence on serum cholesterol by other components of the MFGMs such as, for example, phospholipids, was not excluded [45]. Recently, Noh and Koo demonstrated that sphingomyelin is an effective inhibitor of intestinal absorption of cholesterol in rats [27].

Our aim was to analyze MFGM protein expression-profiles in two breeds, Holstein and Chianina, representing dairy and beef types respectively, by utilizing Two-Dimentional Isolectrofocusing- Sodium dodecyl sulfate polyacrylamide gel electrophoresis (2D IEF-SDS PAGE). Our proteomic study provides molecular insights into the physiological differences between Holstein and Chianina cattle breeds. 

The proposed method analyzes udder indirectly, by extracting mammary epithelial cells of the lactating cow from milk. This bypasses the need for biopsies or animal slaughtering, a limiting factor in sampling, for both ethical and economic reasons. 

## 2. Results and Discussion

**Figure 1:**
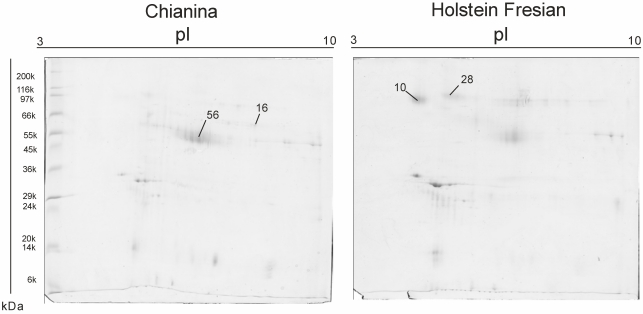
2D Isolectrofocusing-Sodium dodecyl sulfate polyacrylamide gel electrophoresis of MFGM protein fractions of Chianina and Holstein cattle, respectively. Protein spots reported in Table 1 are indicated.

Mammary epithelial cells were successfully isolated from milk samples, resulting in a suitable system to avoid the sampling of udders by biopsy or from animal slaughtering. In 2D IEF-SDS PAGE maps, differences in protein contents between the two breeds can be observed. 2D maps show that we successfully reduced the casein amount, so that less abundant proteins such as MFGM proteins become visible, and that extraction of MFGM proteins was successful. Representative maps are shown in figure 1. Results are reported in Table 1.

Semiquantitative analysis shows an increase of glycoprotein antigen MGP57/53 (milk fat globule epidermal growth factor homologue) in Chianina versus Holstein. MGP57/53 is supposed to be the bovine analogue of mouse Milk Fat Globules-Epidermal Growth Factor 8 (MFG-E8) protein, associated with cell apoptosis [53]. Hayanama and Nagata [54] show that the expression of MFG-E8 in mice mammary gland is strongly upregulated when mammary gland undergoes involution. Primary epithelial cells from involuting mammary gland express MFG-E8. Moreover, macrophages present in the mammary gland at the late stage of involution also express MFG-E8. It should be pointed out that in MFG-E8 knockout mice mammary gland involution has been shown to be severely impaired. Moreover, after the mice started to nurse the second litter, the lobular-alveolar structure was not properly developed [55]. MFG-E8 expression in mouse mammary glands has been shown to be upregulated after parturition and maintained overexpressed during lactation even at a late stage [56]. A recent analysis of gene expression in mouse mammary gland involution, conducted by microarray technique, indicated that MFG-E8 transcripts gradually increased to about 1.5 times in the normalized intensity within three days after forced weaning in l0-days lactating mice [57]. Experimental data suggest that MFG-E8 might also be involved in the recognition and clearance of apoptotic mammary epithelial cells during involution [44]. Two independent groups reported that MFG-E8 is a critical protein for mammary gland remodelling during involution in MFGE8 knockout mice. A deficiency in MFG-E8 caused delayed clearance of apoptotic mammary epithelial cells as well as impaired involution and inflammation of the mammary gland [58,59,60,61,62,63]. 

**Table 1:** Differences in MFGM fractions, Chianina *vs.* Holstein breed. Spot number (SSP), protein molecular weight (Mw, kDa), isoelectric point (PI), number of peptides successfully identified by mass spectrometry (No. peptides), mascot score, protein accession numbers, protein ID and variation fold.

SSP	Mw. kDa	PI	No. peptides	Mascot Score	NCBI accession number	Protein ID	Fold of Variation (C/F)
10	60.734	4.69	10	669	gi|115495209	zymogen granule membrane glycoprotein 2 (GP2) [Bos taurus]	−0.27854
16	45.704	8.59	13	987	gi|2136760	adipocyte differentiation-related protein (ADRP) [Bos taurus]	0.39175
28	83.695	7.07	8	635	gi|3914346	Polymeric immunoglobulin receptor (PIGR)	−0.35186
56	45.704	7.07	16	987	gi|2136760	glycoprotein antigen MGP57/53. mammary gland - bovine	0.28378

This could suggest that MFG-E8 might have a role in the different structure of the Chianina mammary gland, smaller in size compared to the Holstein, and not specialized for large-scale milk production.

Chianina samples also show a higher expression of the adipocyte differentiation-related protein (ADRP). This protein, previously believed to be specific for adipocytes, is a major constituent of the globule surface and is present in a complex containing stoichiometric amounts of xanthine oxidase and butyrophilin. ADRP is one of the earliest markers of adipocyte differentiation [64,65]. The mRNA for ADRP rises at least 100-fold within the first day after the induction of differentiation in cultured preadipocytes, before the increase of fatty acid-binding protein and lipoprotein lipase, early markers of adipocyte differentiation, can be observed [66]. Although the function of ADRP remains unknown, its expression in milk-secreting cells provides the suggestion that it might be involved in the deposition of triacylglycerols droplets in the cytosol, a phenomenon known to occur in both adipocytes and in mammary epithelial cells. In mammary cells, ADRP seems to be concentrated specifically in cellular components involved in the generation of MFG. The hydrophobic *N*-terminus of ADRP, coupled with an apparent covalent of fatty acids, appear to allow its interaction with lipid droplets. Indeed, myristoylation and palmitoylation of hydrophobic peptides seem to increase the partitioning of the peptides into lipid micelles in model systems. Because ADRP is apparently synthesized without a signal peptide, and lacks a transmembrane sequence [64], the secretion of this protein cannot be predicted. Its secretion may be due to its interaction with the milk lipid droplets. In both adipocytes [67] and milk-secreting cells [68], triacylglycerol-rich droplets are formed in the endoplasmic reticulum membrane, and the surface coat on forming droplets in both cell types seems to be contiguous with endoplasmic reticulum membrane. Recently, it has been shown that treatment of rats with an inhibitor of carnitine palmitoyltransferase I induces the expression of ADRP in liver [69]. Lipid droplets also accumulated in livers of etomoxir-treated rats. This result suggests that ADRP could be involved in lipid deposition into droplets. ADRP might be an adaptor molecule involved in interaction between the droplet surface and the butyrophilinxanthine oxidase-containing inner membrane coat of apical plasma membrane [70]. The differences in MFGM proteins involved in lipid deposition that we observed in our samples, could suggest a different lipid composition in the milk of the two breeds, which deserves to be further investigated 

Polymeric immunglobulin receptor-like protein (PIGR) is more expressed in Holstein MFGM samples. PIGR is expressed on several glandular epithelia, including those of liver and breast. It is a member of the immunoglobulin superfamily which mediates transcellular transport of polymeric immunoglobulin molecules. The receptor is composed of five units with homology to the variable (V) units of immunoglobulins and a transmembrane region, which also has some homologies to the immunoglobulin variable region [70]. Recently, Ng *et al.* [71] tested milk protein effectiveness against HIV-1 proteins. It has been shown that *PIGR* inhibits HIV-1 protease and HIV-1 integrase, with a strenght similar to that of lactoferrin. PIGR is present at a concentration exceeding 0.2 M (at the end of the isolation procedure) in bovine milk, while its inhibitory concentration (IC50) value for HIV-1 reverse transcriptase activity is 4.8 M. Hence it exerts some inhibitory effect on the HIV-1 enzyme at the physiological concentration found in bovine milk [72].

Glycoprotein 2 (zymogen granule membrane, GP2) is overexpressed in Holstein. GP2 is the major membrane protein present in the pancreatic zymogen granule, it is cleaved and released into the pancreatic duct along with exocrine secretions and has been reported to be expressed in milk MFGM [70]. Yu and Lowe showed that GP2 binds *E. coli* expressing Type 1 fimbria, and that binding is dependent on GP2 glycosylation, and specifically on the presence of mannose residues [73]. GP2, when binding to Type I fimbriae, may serve as a physical barrier and as a molecular decoy for bacterial adhesion. Although the common bile duct and pancreatic duct share a common exit to the intestine, ascending infections of the pancreatic duct are not reported in the literature and are not commonly observed in the clinical setting, even in chronic pancreatitis, when the pancreatic duct is obstructed. In contrast, ascending biliary tract secondary infections are relatively common. Noticeably, GP2 comprises most of the protein precipitate present in the duct of patients afflicted with chronic pancreatitis, and thus may serve as a protective against infections [74].

## 3. Experimental Procedure

*Samples:* Fresh milk samples of individuals belonging to breeds of different origins and selected for different purposes (beef, Chianina, and dairy, Holstein) were collected. Chianina is the largest and one of the oldest beef cattle breeds in the world. Holstein is the highest producing dairy breed in the world. Samples from two animals per breed (two replicates per breed) were collected exactly at the same lactation phase, seven days after calving, and transported in ice to the laboratory. All individuals were raised on the same farm under the same conditions.

*MFGM extraction*: Bovine MFGM was isolated by a procedure in which the fat globules were separated from whole milk and washed several times with physiological buffers to lay down caseins. The membrane was then released from the surface of the globules by physical or chemical means and collected by centrifugation [75,76,77,78,79]. Five hundred mL of milk were centrifuged at 2,000*g* for 30 min at 4 °C to remove cells, obtaining cream. The recovered cream layer was washed five times with 7.4 pH Phosphate Saline Buffer solution to remove caseins. Washed globules were stored at −20°C until used. To extract the MFGM proteins, washed cream was mixed 1:3 with an SDS-containing solution (7 M urea, 2 M thiourea, 4% CHAPS, 1% Triton X-100, 20 mM Tris, 1% DTT and 0.5% IPG buffer) following Quaranta *et al*. [80], incubated in ice for 60 min and periodically vortexed, then centrifuged at 10,000 g for 1 hour. After removing the floating cream layer, the supernatant was subjected to precipitation with methanol and chloroform following Wessel *et al.* [81]. Before focusing, the sample was incubated for 3 h at room temperature, under strong agitation, to perform alkylation with 7.7 mM Iodoacetamide in a solution of 7 M urea, 2 M thiourea, 4% CHAPS, 20 mM Tris, pH 3–10 carrier ampholyte, 40 mM Tris, 5 mM TBP, 0.1 mM EDTA (pH 8.5), 2% (v/v) protease inhibitor cocktail (Sigma-Aldrich). To prevent over-alkylation, iodoacetamide excess was destroyed by adding equimolar amount of DTE.

*Semiquantitative IEF-SDS PAGE*: IEF was performed using ready-to-use Immobiline Dry-Strips linear pH gradient 3–10 length 18 cm (BioRad, CA, USA) and the in gel sample rehydration method. 600 µg of proteins per strip were loaded. IEF was run on a BioRad Protean IEF at 20 °C constant temperature and 8,000 V for 99,000 Vh. After IEF, the IPG gel strips were incubated at room temperature for 30 min in 6 M urea, 30% w/v glycerol, 2% w/v SDS, 5 mM Tris-HCl, pH 8.6. The strips were sealed at the top of a 1.0 mm vertical second dimensional gel (BioRad) with 0.5% agarose in 25 mM Tris, 192 mM glycine, 0.1% SDS, pH 8.3. SDS-PAGE was carried out on homogeneous running gels 12 % T 3% C. The running buffer was 25 mM Tris, 192 mM glycine, 0.1% SDS, pH 8.3 and running conditions were 40 mA/gel until the bromophenol blue reached the bottom of the gel. Molecular weight marker used was Wide Range SigmaMarker^TM^ (BioRad). Gels were automatically stained with Brilliant Blue G colloidal (Sigma, St. Louis, MO, USA) following the manufacturer’s instructions. Four technical replicates per sample were performed, for a total of 16 gels. The 2-DE image analysis was carried out and spots were detected and quantified using the Progenesis SameSpots software v.2.0.2733.19819 software package (Nonlinear Dynamics, Newcastle UK). Each gel was analysed for spot detection and background subtraction. Within-group comparison of protein spot numbers was determined by repeated measure analyses. Among-group comparisons were determined by ANOVA (Analysis of Variance) procedure in order to classify sets of proteins that showed a statistically significant difference with a confidence level of 0.05.

*In-Gel Digestion*: Spots from 2-DE maps were carefully excised from the gel and subjected to in-gel trypsin digestion according to Shevchenko *et al.* [82] with minor modifications. The gel pieces were swollen in a digestion buffer containing 50 mM NH_4_HCO_3_ and 12.5 ng/mL trypsin (modified porcine trypsin, sequencing grade, Promega, Madison, WI, USA) in an ice bath. After 30 min, the supernatant was removed and discarded; then 20 mL of 50 mM NH_4_HCO_3_ were added to the gel pieces, and digestion was allowed to proceed overnight at 37 °C. The supernatant containing the peptide mixture was removed and acidified with 5% formic acid before injection in the mass spectrometer.

*Protein identification by MS/MS*: Peptide mixtures were separated using Ultimate-Switchos-Famos HPLC system (LC Packings, Amsterdam, The Netherlands). A sample volume of 14 µL was loaded by the autosampler onto a homemade 2 cm fused silica precolumn (75 µm I.D.; 375 µm O.D) Reprosil C18-AQ, 3 µm (Ammerbuch-Entringen, DE) at a flow rate of 2 µL/min [83]. Sequential elution of peptides was accomplished using a flow rate of 200 nL/min and a linear gradient from Solution A (2% acetonitrile; 0.1% formic acid) to 50% of Solution B (98% acetonitrile; 0.1% formic acid) in 40 minutes over the precolumn in-line with a homemade 10-15 cm resolving column (75 µm I.D.; 375 µm O.D.; Reprosil C18-AQ, 3 µm, Dr. Maisch GmbH, Ammerbuch-Entringen, Germany). Peptides were eluted directly into a High Capacity ion Trap HCTplus (Bruker-Daltonik, Bremen, Germany). Capillary voltage of 1.5–2 kV and a dry gas flow rate of 10 L/min were used at a temperature of 230 °C. The scan range used was from 300 to 1800 m/z. Protein identification was performed by searching in the National Center for Biotechnology Information non-redundant database (NCBInr, version 20081128, www.ncbi.nlm.nih.gov) using the Mascot program in-house version 2.2 (Matrix Science, London, UK). The following parameters were adopted for database searches: complete carbamidomethylation of cysteines and partial oxidation of methionines, peptide Mass Tolerance ±1.2 Da, Fragment Mass Tolerance ±0.9 Da, missed cleavages 2. Only result scores [−10 x Log(P)] over the significance threshold level (P < 0.05) were identified as positive. Even when high MASCOT scores were obtained (values > 60), if proteins were identified by a single peptide a combination of automated database search and manual interpretation of peptide fragmentation spectra was used to validate protein assignments. The mass error, the presence of fragment ion series and the expected prevalence of C-terminus containing ions (Y-type) in the high mass range were all taken into account in the verification procedure. Moreover, protein hit identity was confirmed by replicate measurements.

## 4. Conclusions

Using a proteomic approach, we have successfully identified some of the major MFGM proteins in two different cattle breeds. We utilised a nonbiased method for the discovery of specific molecules involved in this unique secretory function. This work provides a preliminary study of the complexity of the secretion of MFG in mammary epithelial cells during the lactation process. 

A considerable advantage of the proposed method relies on the possibility of analysing this peculiar kind of cells without the need of biopsy or animal slaughtering, which often represents a limiting factor in sampling, for both ethical and economic reasons. 

We analysed samples of two cattle breeds selected for different purposes, and our preliminary results indicate that protein differences at the MGF level can be observed even if the animals were raised in the same conditions. In detail, PIGR, having a role in the immunoglobulin function, and GP2, a protein that may serve as a protective against infections, resulted overexpressed in Holstein, which is a highly specialised dairy breed. Dairy breeds are selected, among other traits, for resistance to mastitis and for somatic cell score. This may represent an effect of long time selection and could lead on an insight on the effects of milk of different origin on calf health and on its nutraceutical characteristics. In Chianina beef breed ADRP, a protein involved in lipid deposition, is overexpressed. Further studies should focus on fat amount and composition in the milk of this breed, and on the potential implications of such information for the human diet. Also MFG-E8, a protein involved in the lactation process and showing anti-viral activity, is overexpressed in this breed at the lactation stage analysed. Beyond having antiviral properties, MFG-E8 could be related to the different timing in mammary gland maturation and drying off between the two breeds. 

Further investigations are needed to better define the difference in MFGM protein composition between Holstein and Chianina breeds and to fully describe the variation of the MFGM proteins during the lactation phases.

Our study shows that proteomics is a suitable method for the investigation of the characteristics of milk proteins. Moreover, it is a powerful tool in nutritional genomics, allowing the analysis of milk as food and helping the selection of animals with characteristics relevant for human health.

## Acknowledgments

This work was financially supported by the “GENZOOT” research program funded by the Italian Ministry of Agriculture. We wish to thank Stefano Corbianco for providing the samples, Maria Giulia Egidi for help in mass spectrometry analysis and Paolo Ciorba for sampling assistance. 
